# Ablation of Whirlin Long Isoform Disrupts the USH2 Protein Complex and Causes Vision and Hearing Loss

**DOI:** 10.1371/journal.pgen.1000955

**Published:** 2010-05-20

**Authors:** Jun Yang, Xiaoqing Liu, Yun Zhao, Michael Adamian, Basil Pawlyk, Xun Sun, D. Randy McMillan, M. Charles Liberman, Tiansen Li

**Affiliations:** 1 The Berman-Gund Laboratory for the Study of Retinal Degenerations, Harvard Medical School, Massachusetts Eye and Ear Infirmary, Boston, Massachusetts, United States of America; 2 Department of Pediatrics, University of Texas Southwestern Medical Center, Dallas, Texas, United States of America; 3 Department of Otology and Laryngology, Harvard Medical School and Eaton-Peabody Laboratory, Massachusetts Eye and Ear Infirmary, Boston, Massachusetts, United States of America; University of Massachusetts Medical School, United States of America

## Abstract

Mutations in whirlin cause either Usher syndrome type II (USH2), a deafness-blindness disorder, or nonsyndromic deafness. The molecular basis for the variable disease expression is unknown. We show here that only the whirlin long isoform, distinct from a short isoform by virtue of having two N-terminal PDZ domains, is expressed in the retina. Both long and short isoforms are expressed in the inner ear. The N-terminal PDZ domains of the long whirlin isoform mediates the formation of a multi-protein complex that includes usherin and VLGR1, both of which are also implicated in USH2. We localized this USH2 protein complex to the periciliary membrane complex (PMC) in mouse photoreceptors that appears analogous to the frog periciliary ridge complex. The latter is proposed to play a role in photoreceptor protein trafficking through the connecting cilium. Mice carrying a targeted disruption near the N-terminus of whirlin manifest retinal and inner ear defects, reproducing the clinical features of human USH2 disease. This is in contrast to mice with mutations affecting the C-terminal portion of whirlin in which the phenotype is restricted to the inner ear. In mice lacking any one of the USH2 proteins, the normal localization of all USH2 proteins is disrupted, and there is evidence of protein destabilization. Taken together, our findings provide new insights into the pathogenic mechanism of Usher syndrome. First, the three USH2 proteins exist as an obligatory functional complex in vivo, and loss of one USH2 protein is functionally close to loss of all three. Second, defects in the three USH2 proteins share a common pathogenic process, i.e., disruption of the PMC. Third, whirlin mutations that ablate the N-terminal PDZ domains lead to Usher syndrome, but non-syndromic hearing loss will result if they are spared.

## Author Summary

Usher syndrome is a devastating genetic disorder affecting both vision and hearing. It is classified into three clinical types. Among them, type II (USH2) is the predominant form accounting for about 70% of all Usher syndrome cases. Three genes, *USH2A*, *USH2C*, and *USH2D*, underlie the development of USH2; and they encode usherin, Very Large G protein-coupled Receptor-1 (VLGR1), and whirlin, respectively. In this study, we show that the long whirlin isoform organizes the formation of a multi-protein complex *in vivo* that includes usherin and VLGR1. Targeted disruption of whirlin long isoform abolishes the normal cellular localization of the two partner USH2 proteins in the retina and in the inner ear and causes visual and hearing defects. We present the first definitive evidence that the USH2 proteins mark the boundary of the periciliary membrane complex, which was first described in frog photoreceptors and is thought to play a role in regulating intracellular protein transport. We propose that defects in all USH2 proteins share a common pathogenic pathway by disrupting the periciliary membrane complex in photoreceptors.

## Introduction

Usher syndrome manifests as both retinal degeneration and hearing loss [1], [2]. It is classified into type I, II, and III based on clinical features of the hearing defects [3]–[8]. Usher syndrome type I (USH1) presents with profound congenital deafness and vestibular dysfunction. USH2 is the most common form and is characterized by moderate non-progressive hearing loss without vestibular dysfunction. USH3 is distinguished from USH2 by the progressive nature of its hearing loss and occasional vestibular dysfunction. There is further genetic heterogeneity within each clinical type of Usher syndrome. For example, three distinct gene loci, referred to as *USH2A*, *USH2C* and *USH2D*, are known to underlie USH2. These three genes encode the USH2A protein (also known as usherin), Very Large G protein-coupled Receptor-1 (VLGR1) and whirlin, respectively. Among these, mutations in *USH2A* account for over 70% of USH2 patients whereas *USH2C* and *USH2D* are responsible for the remainder. A previously proposed *USH2B* locus was subsequently shown to be in error and has been withdrawn [9].

**Figure 1:**
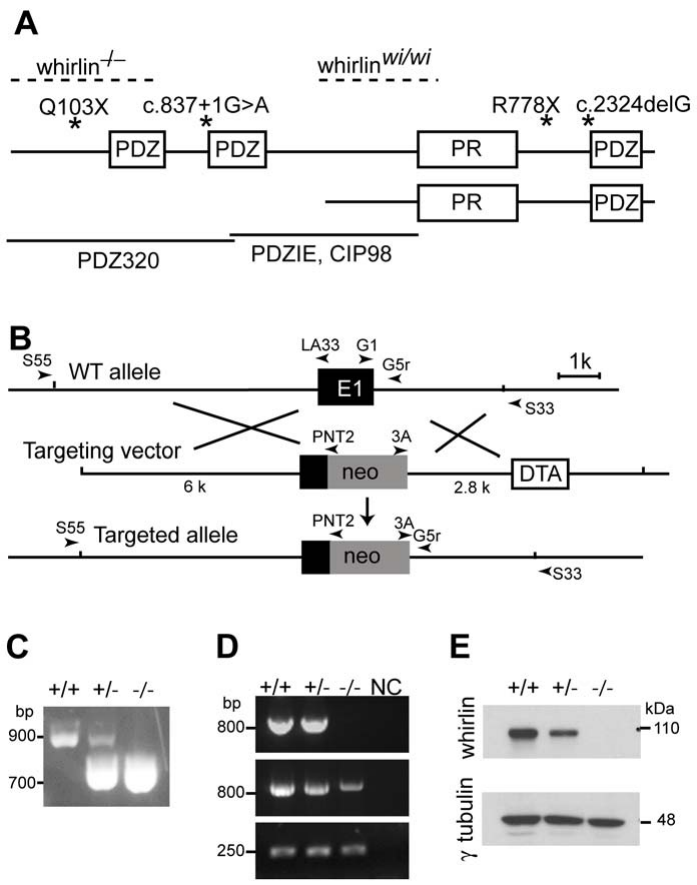
Whirlin knockout mice were generated. (A) A schematic diagram illustrating the long and short isoforms of whirlin. The dashed lines indicate the deletion regions of the whirlin gene in whirlin knockout (whirlin^−/−^) and whirler (whirlin^wi*/wi*^) mice. The asterisks indicate the mutations of the whirlin gene in humans. The bottom solid lines indicate the antigen regions of various whirlin antibodies. PDZ, postsynaptic density 95/discs large/zonula occludens 1; PR, proline-rich region. (B) Targeting strategy for disrupting the whirlin gene. PCR primers for identification of the mutant and wild-type alleles are shown as arrowheads. E1, exon 1; neo, neomycin, the positive selective marker; DTA, diphtheria toxin expression cassette, the negative selective marker. (C) Identification of the mutant allele by genomic PCR using primers G1, G5r and 3A. (D) RT-PCR analysis shows loss of the first exon of whirlin transcripts in the homozygous mutant retina. Whirlin mRNA transcripts were reverse transcribed and amplified using primers located on exon 1 and 6 (top panel), exon 2 and 6 (middle panel), and exon 11 and 12 (bottom panel). Exon 12 is the last exon of the whirlin gene. NC, negative controls with water instead of DNA samples. (E) The whirlin long isoform was completely knocked out in the retina of homozygous mutants as shown by immunoblotting. γ–tubulin served as a loading control. +/+, wild-types; −/−, whirlin knockout homozygotes; +/−, whirlin knockout heterozygotes.

Genetic defects in the whirlin gene have long been known as a cause of nonsyndromic deafness DFNB31 [10], [11] and, more recently, were found to underlie USH2D [12]. Whirlin R778X and c.2423delG mutations (Figure 1A) that truncate the protein close to its C-terminus cause profound prelingual hearing impairment in humans. In the naturally occurring whirler mouse, from which the name whirlin was derived, a large deletion was found in the middle of the whirlin gene (Figure 1A). Similar to human patients with DFNB31, the whirler mouse suffers from inner ear defects [10]. Neither patients with DFNB31 nor the whirler mouse manifest any retinal deficits. The whirlin gene defect underlying USH2D arises from compound heterozygosity of a Q103X mutation and a c.837+1G>A mutation [12], which are positioned in the first and second exon of the whirlin gene, respectively (Figure 1A). Therefore, different mutations of the whirlin gene account for a spectrum of hearing and vision defects although the mechanism underlying the variable disease expression of different mutations in the whirlin gene is not known.

Multiple whirlin transcript variants were found in the inner ear [10], [13], [14]. They are conceptually translated into two groups of proteins, the long and short isoforms (Figure 1A). The whirlin long isoform contains two N-terminal PDZ domains, a proline-rich domain and a third PDZ domain near the C-terminus. Heterogeneity in the whirlin short isoform arises from use of alternative transcriptional start sites and/or splicing sites of the whirlin gene, which generates several variants with different N-termini. The short isoform has no N-terminal PDZ domains but retains the proline-rich region and the third C-terminal PDZ domain. Both the PDZ domain and proline-rich region are modular protein interaction domains. PDZ domains bind to a short conserved sequence, known as a PDZ-binding motif, present at the C-terminus of proteins or found as an internal motif [15]. A proline-rich region usually binds to WW and SH3 domains [16]. With these two types of protein interaction domains, whirlin is believed to be engaged in the assembly of supramolecular complexes at specific subcellular locations. A series of in vitro analyses have found that whirlin is able to interact with usherin [17] and VLGR1 [14], the two causative proteins for other forms of USH2 [18]–[20]. A recent report demonstrates that these interactions probably exist at the ankle-link complex in developing hair cells [21].

A few reports have been published which examined the localization of whirlin in photoreceptors [14], [22], [23]. Whirlin has been reported to localize to the apical inner segment collar, the ciliary apparatus, the adherens junctions and the synaptic region of photoreceptors [14], [23]. However, there is no consensus from these reports on where the whirlin protein is localized in photoreceptors. As the photoreceptors are highly polarized neurons and are well organized into stratified layers of the retina, whether a protein is localized to the apical inner segment vs. the synaptic layer has completely different implication for its putative functions. More importantly, there has been no *in vivo* study of any kind on the association among the three USH2 proteins in photoreceptors. To fill in this knowledge gap, we carried out targeted disruption of the whirlin gene in mice at the 5′-terminal region. This disruption abolishes the long isoform and simulates the human mutations that cause USH2D. This mutant line of mice reiterated the vision and hearing defects of human USH2 patients. Using this mouse line and the *Ush2A* and *Ush2C* mutant mouse lines that had been previously generated, we analyzed the expression, localization and function of whirlin in the retina and compared them with those in the inner ear cochlea. We further analyzed the interaction among the USH2 proteins using those mouse lines as in vivo model systems. Our data provide new insight into the function of whirlin and other USH2 proteins and point to a possible disease mechanism for USH2. The data also help to explain the molecular basis for the variable disease expression caused by mutations in different regions of the whirlin gene.

## Results

### Whirlin knockout mice do not express the whirlin long isoform at the RNA and protein levels

A whirlin mutant mouse line was generated by replacing a portion of exon 1, which included the translation start codon for the whirlin long isoform, with a Neo^r^ expression cassette (Figure 1B). The targeted allele was confirmed by amplifying the genomic DNA fragments containing the junctional sequences between the whirlin gene and the Neo^r^ expression cassette. To determine if expression of whirlin was abolished in the mutant mice, we conducted RT-PCR and western blotting analyses in the retina. RT-PCR analysis verified that the first exon of whirlin transcripts was absent in the homozygotes (Figure 1D). Western blotting analysis showed that the whirlin long isoform, normally migrating at an apparent molecular weight of about 110 kDa, was completely absent in the retina of homozygous mice (Figure 1E). Thus, this targeted allele of whirlin is a null allele for the whirlin long isoform. To distinguish it from the existing whirler mice, we refer to this line of mutant mice as the whirlin knockout mouse. Whirlin knockout mice appeared viable and comparable to their wild-type littermates in growth characteristics, reproductive performance and general health.

### The whirlin long isoform, but not the short isoform, is expressed in the retina

**Figure 2:**
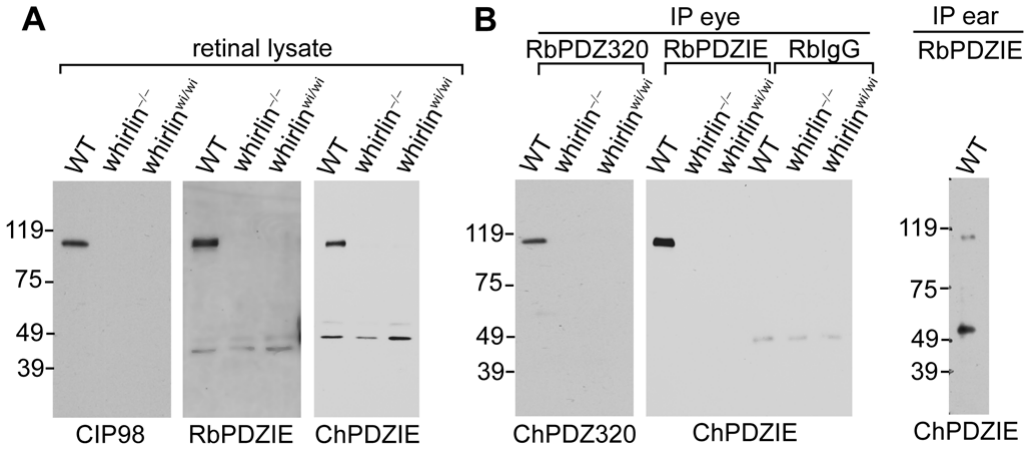
Whirlin expresses its long isoform in the retina. (A) Western blotting analysis of retinal lysates. All CIP98, RbPDZIE, and ChPDZIE antibodies detected only the whirlin long isoform. The lower bands on the blots of RbPDZIE and ChPDZIE are nonspecific, because the bands on these two blots have different molecular weights and they are present in the retina of whirlin knockout and whirler mice. (B) Western blotting of immunoprecipitates from the retina (left) and the inner ear (right). The ChPRZIE antibody detected only the whirlin long isoform in the RbPDZIE immunoprecipitate from the retina, but detected both the long and short isoforms from the inner ear. The ChPDZ320 antibody detected only the whirlin long isoform but not an N-terminal whirlin fragment in the RbPDZ320 immunoprecipitate from the retina. RbPDZIE and ChPDZIE, the rabbit and chicken PDZIE (Figure 1A) antibody, respectively; RbPDZ320 and ChPDZ320, the rabbit and chicken PDZ320 (Figure 1A) antibody, respectively; RbIgG, rabbit immunoglobulin, a negative control. WT, wild-types; whirlin^−/−^, whirlin knockout mutants; whirlin*^wi/wi^*, whirler mutants.

To examine the normal expression of whirlin isoforms at the protein level in the retina, we generated a series of antibodies against whirlin and used two whirlin mutant mouse lines, whirlin knockout and whirler mice, as negative controls. In whirlin knockout mice, deletion of the first exon ablates the long isoform, while mutation in whirler mice eliminates the short isoform [10] (Figure 1A). Rabbit PDZIE, chicken PDZIE, and CIP98 [24] antibodies are directed against epitopes common in both the whirlin long and short isoforms (Figure 1A). Western blotting using these antibodies detected only the whirlin long isoform in the wild-type (WT) retina (Figure 2A), suggesting that the short isoform was either not expressed or was expressed at such a low level that was beneath the threshold of detection by this assay. To confirm this result, we enriched the whirlin protein(s) from the retinal lysate by immunoprecipitation using the rabbit PDZIE antibody, and then performed western blotting analysis of the precipitates using the chicken PDZIE antibody. While we found significant enrichment of the whirlin long isoform, we again did not detect the short isoform. As a positive control, we found both isoforms were enriched and readily detectable in the cochlear immunoprecipitate (Figure 2B). Therefore, the whirlin short isoform in the retina is a rare variant if expressed at all. In addition to the long and short variants reported previously, we found a distinct N-terminal transcript of whirlin in the retina by screening a mouse retinal cDNA library. This transcript terminates in the middle of the second PDZ domain such that if translated, it would produce a whirlin protein that includes only the first N-terminal PDZ domain. This transcript is therefore not affected by the whirler mutation or by the corresponding human mutations causing DFNB31 (Figure 1A). To examine whether this N-terminal whirlin isoform was abundant at the protein level in the retina, we performed immunoprecipitation using the rabbit PDZ320 antibody, whose antigen is the N-terminal 320 amino acids of whirlin (Figure 1A). Again only the whirlin long isoform was detected by western blotting using the chicken PDZ320 antibody (Figure 2B), suggesting this N-terminal whirlin isoform is not an abundant variant either. Nevertheless the presence of an N-terminal whirlin variant may be of functional significance. Taken together, these results clearly demonstrate that the whirlin long isoform is the predominant variant expressed in the retina.

### Whirlin is localized at the periciliary membrane complex (PMC) in mouse photoreceptors

**Figure 3:**
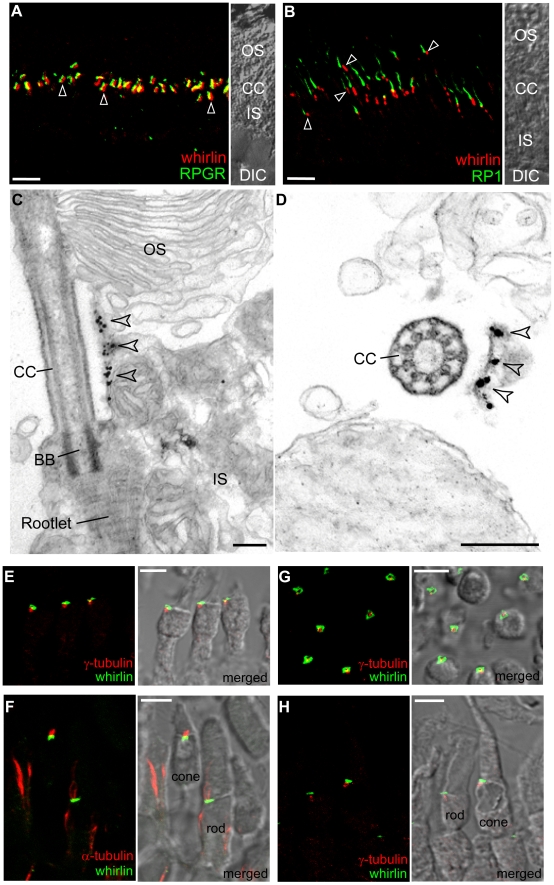
Whirlin is localized at the PMC and at the PRC. Whirlin is localized at the PMC in mouse photoreceptors (A–D) and at the PRC in frog photoreceptors (E–H). (A) Whirlin (red, arrowheads) was localized closely next to the connecting cilia, marked by RPGR (green), between the inner and outer segments in the mouse retina. The corresponding phase contrast image is attached on the right. (B) Whirlin (red, arrowheads) was localized obliquely below the signals of RP1 (green) in mouse photoreceptors. The corresponding phase contrast image is attached on the right. (C,D) Gold labels of whirlin (arrowheads) are present on the plasma membrane of inner segments facing the connecting cilia as shown by the longitudinal (C) and transverse (D) views of immunoelectron microscopy. (E–H) Whirlin (green) was localized above the basal bodies marked by γ-tubulin (red, E,H) and below the axonemal microtubules marked by acetylated α-tubulin (red, F) in the longitudinal views of frog photoreceptors. It appears as circles surrounding the basal bodies in the transverse view (G). The distribution of whirlin is same in both rod and cone photoreceptors (F,H). The merged images on the right (E–H) are superimposed immunofluorescent images and their corresponding phase contrast images. OS, outer segments; CC, connecting cilia; IS, inner segments; DIC, differential interference contrast image; BB, basal body. Scale bars, 5 µm (A,B,E–H), 200 nm (C,D).

Photoreceptors are highly polarized sensory neurons consisting of three major subcellular compartments, the outer segment, the inner segment and the synaptic terminus. Linking the light sensing outer segment and the biosynthetic inner segment is a thin bridge known as the connecting cilium. By immunofluorescence whirlin was found at the vicinity of the connecting cilia (Figure 3A and 3B) but not in the photoreceptor synaptic layer (the outer plexiform layer, data not shown). RPGR (retinitis pigmentosa GTPase regulator) and RP1 (retinitis pigmentosa 1) are proteins known to be localized at the connecting cilia and at the axonemal microtubules distal to the connecting cilia, respectively [25] (see Discussion). Double staining of whirlin with either RPGR or RP1 showed whirlin to localize adjacent to RPGR (Figure 3A) but proximal to RP1 (Figure 3B). However, unlike RPGR, RPGRIP1 and other ciliary proteins, immunostaining of dissociated photoreceptors, which include the outer segments and the connecting cilia, could not detect any whirlin signals at the connecting cilia (data not shown). This indicated that whirlin was not a core component of the connecting cilia. Immunoelectron microscopy from both longitudinal (Figure 3C) and cross (Figure 3D) sections found the immunogold labels of whirlin at a plasma membrane microdomain in the apical inner segment, which wraps around the connecting cilium and is usually destroyed in the dissociated photoreceptors. Thus, data from immunofluorescent staining and immunoelectron microscopy were consistent with whirlin localizing to a membrane microdomain that surrounds the connecting cilia, a location that is identical to that of usherin [26] (see Discussion).

The distribution pattern of whirlin in mouse photoreceptors was reminiscent of a structure called the periciliary ridge complex (PRC) found in frog photoreceptors [27]. The PRC was defined by a morphological feature, which includes a set of ridges and grooves with a nine-fold symmetry, seen by scanning electron microscopy. It marks a specialized domain on the plasma membrane of the inner segment that surrounds the base of the connecting cilium. To examine whether whirlin was localized at this structure in frogs, we generated an antibody against the C-terminus of frog whirlin. Double staining of whirlin with γ–tubulin and acetylated α–tubulin, markers of basal bodies and axonemal microtubules, respectively, showed that whirlin was localized immediately above the basal bodies (Figure 3E) and beneath the axonemal microtubules (Figure 3F). This is similar to the findings in mouse photoreceptors. In a cross sectional view, the signals of whirlin appeared as circles surrounding the basal bodies (Figure 3G). The diameter of these circles was approximately 2 µm, which is in the range of the previously determined diameter of the PRC [27]. Both rod and cone photoreceptors had the same distribution of whirlin (Figure 3F and 3H). These data suggest that whirlin is a resident protein at the PRC in frogs. The PRC as a morphologically distinct structure is not present in mammalian photoreceptors [28]. However, the conserved whirlin distribution in frog and mouse photoreceptors suggests that a functionally equivalent structure, delineated by the presence of whirlin, exists in the latter. We refer to this PRC-homologous membrane microdomain as the periciliary membrane complex (PMC). Thus, whirlin is a marker of the mammalian photoreceptor PMC.

### Whirlin assembles a multi-protein complex with two known USH2 proteins at the PMC

**Figure 4:**
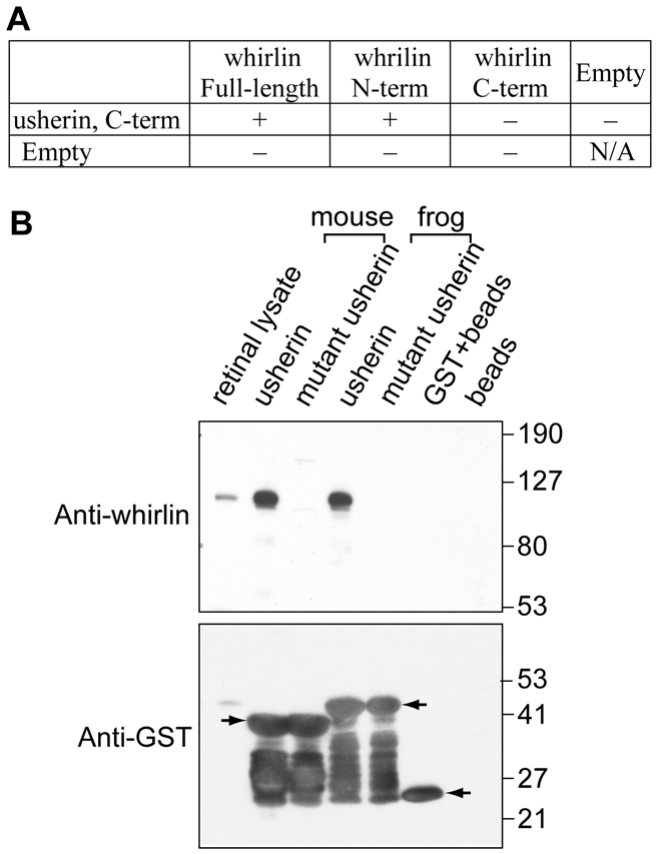
Whirlin directly interacts with usherin. (A) A yeast two-hybrid analysis demonstrates that mouse whirlin, through its N-terminal two PDZ domains (whirlin N-term), interacts with the C-terminal fragment of mouse usherin. The “+” sign denotes growth of large, white yeast colonies and “−” denotes absence of colonies. This experiment was conducted in two orientations. (B) A GST pull-down assay demonstrates that the intact C-terminal fragments of mouse and frog usherin, but not the mutant ones, which lack the PDZ-binding motif, were able to pull down the endogenous whirlin from the retinal lysate, indicating that the PDZ-binding motif of usherin is involved in the interaction between whirlin and usherin. The arrows on the GST blot indicate the positions of mouse and frog GST-fused usherin fragments and GST. The multiple bands below the mouse and frog GST-fused usherin fragments are their degraded products.

The distribution of whirlin in photoreceptors was similar to that of USH2A protein (usherin), which was previously reported by our laboratory [26]. Usherin is predicted to have a PDZ-binding motif at its C-terminus [19]. We investigated whether whirlin and the cytoplasmic C-terminus of usherin interacted with each other. Yeast two-hybrid analysis demonstrated their interaction and the involvement of the first and second PDZ domains of whirlin in this protein binding (Figure 4A). We then sought further confirmation of their interaction by performing GST pull-down assays. We generated frog and mouse usherin-GST fusion protein constructs using either intact or mutant versions of the usherin C-terminal (intracellular) domain. The mutant usherin C-terminal domain lacked a functioning PDZ-binding motif. The expressed GST fusion proteins were incubated with mouse retinal lysate in an attempt to pull down whirlin. The results showed that the intact but not the mutant usherin C-termini were able to pull down endogenous whirlin from retinal lysate (Figure 4B). Therefore, our studies demonstrated that whirlin and usherin directly interacted with each other through the two N-terminal PDZ domains of whirlin and the C-terminal PDZ-binding motif of usherin. Our data support the findings of others reported in recent publications [14], [17].

**Figure 5:**
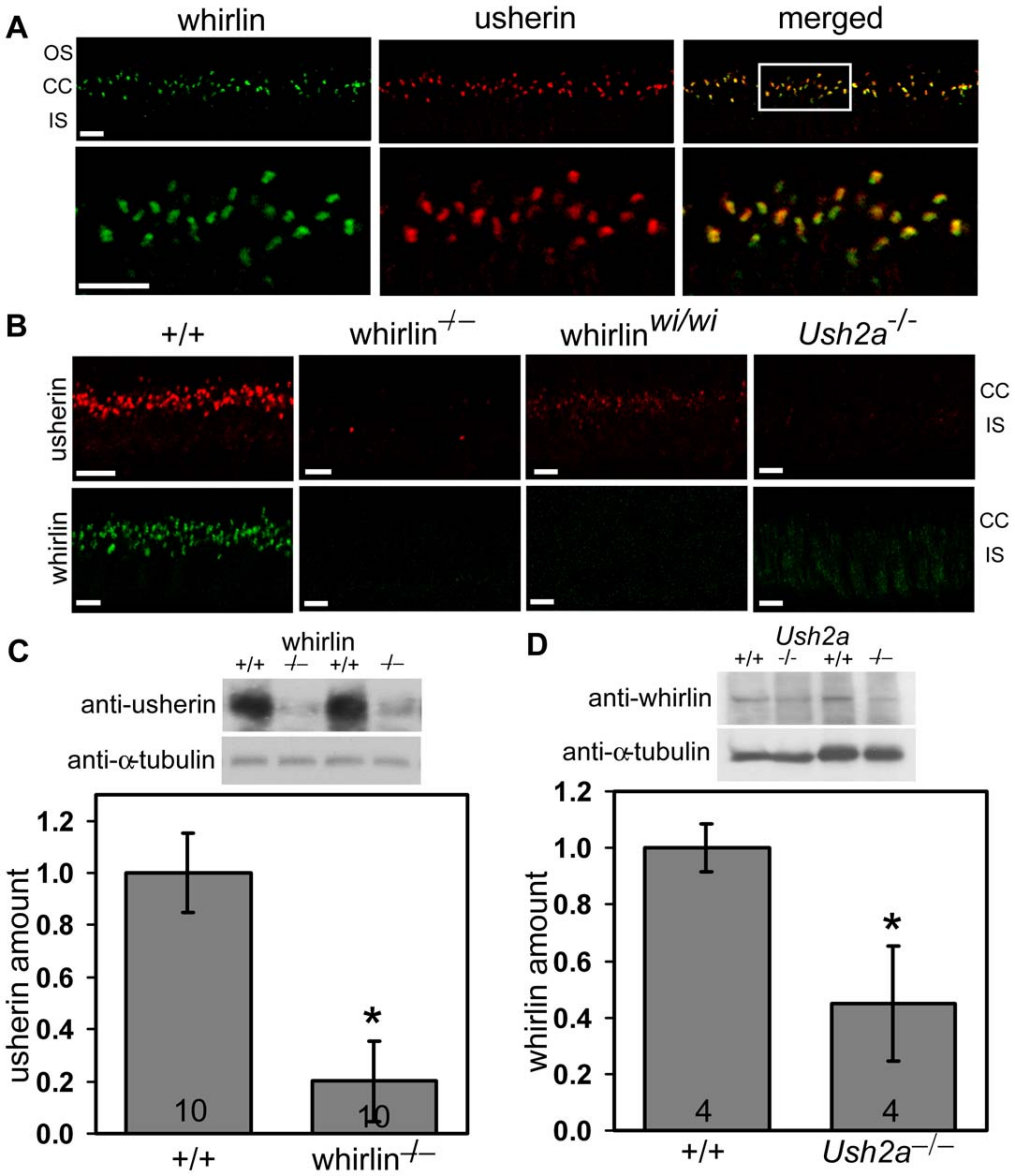
Whirlin and usherin are colocalized in photoreceptors. (A) Whirlin (green) and usherin (red) were colocalized at the PMC in mouse photoreceptors. The bottom panels are the enlarged view of the region marked by the frame in the merged image on the top. (B) The signals of usherin (red, top) and whirlin (green, bottom) were mislocalized from the PMC in whirlin knockout (whirlin^−/−^), whirler mutant (whirlin*^wi/wi^*) and *Ush2a* knockout (*Ush2a^−/−^*) mice (see results section for details). OS, outer segments; CC, connecting cilia; IS, inner segments. Scale bars (A,B), 5 µm. (C) The amount of usherin was reduced by about 80% in the retina of whirlin knockout mice as analyzed by immunoblotting. (D) The amount of whirlin was reduced by about 50% in the retina of *Ush2a* knockout mice as analyzed by immunoblotting. The images on the top of the bar charts in (C,D) are the representative western blots of usherin and whirlin, respectively. The signals of α-tubulin were used as a loading control. Error bars in (C,D) represent the standard error of the mean. The numbers in the bottom of each bar (C,D) are the numbers of animals analyzed.

We next evaluated the *in vivo* interaction between whirlin and usherin by double labeling immunofluorescence. In WT mouse photoreceptors, these two proteins colocalized fully at the PMC (Figure 5A). Examination of their distribution in the retinas of whirlin knockout, whirler and *Ush2a* knockout mice revealed profound perturbation of their localization pattern. In *Ush2a* knockout mice whirlin disappeared from the PMC. In whirlin knockout mice, usherin signals was largely absent from the PMC. In whirler mice, usherin staining was greatly reduced though not extinguished; trace amount of usherin staining was seen uniformly distributed at the PMC of all photoreceptors (Figure 5B). These results suggest that the normal localization of whirlin and usherin at the PMC depends on each other. Thus, ablation of usherin disrupts the normal localization of whirlin, and vice versa. The observation that usherin localization at the PMC was only partially disrupted in whirler mice is consistent with the lack of an overt retinal phenotype in these mice, and can be explained on the basis that the N-terminal PDZ domains of whirlin is not disrupted by the whirler mutation (see Discussion). Loss of binding partners also appeared to destabilize these two proteins. Western blotting analysis showed a reduction in the amount of usherin by 80% in the whirlin knockout mice (Figure 5C), and a reduction in whirlin by 50% in the *Ush2a* knockout mice (Figure 5D).

**Figure 6:**
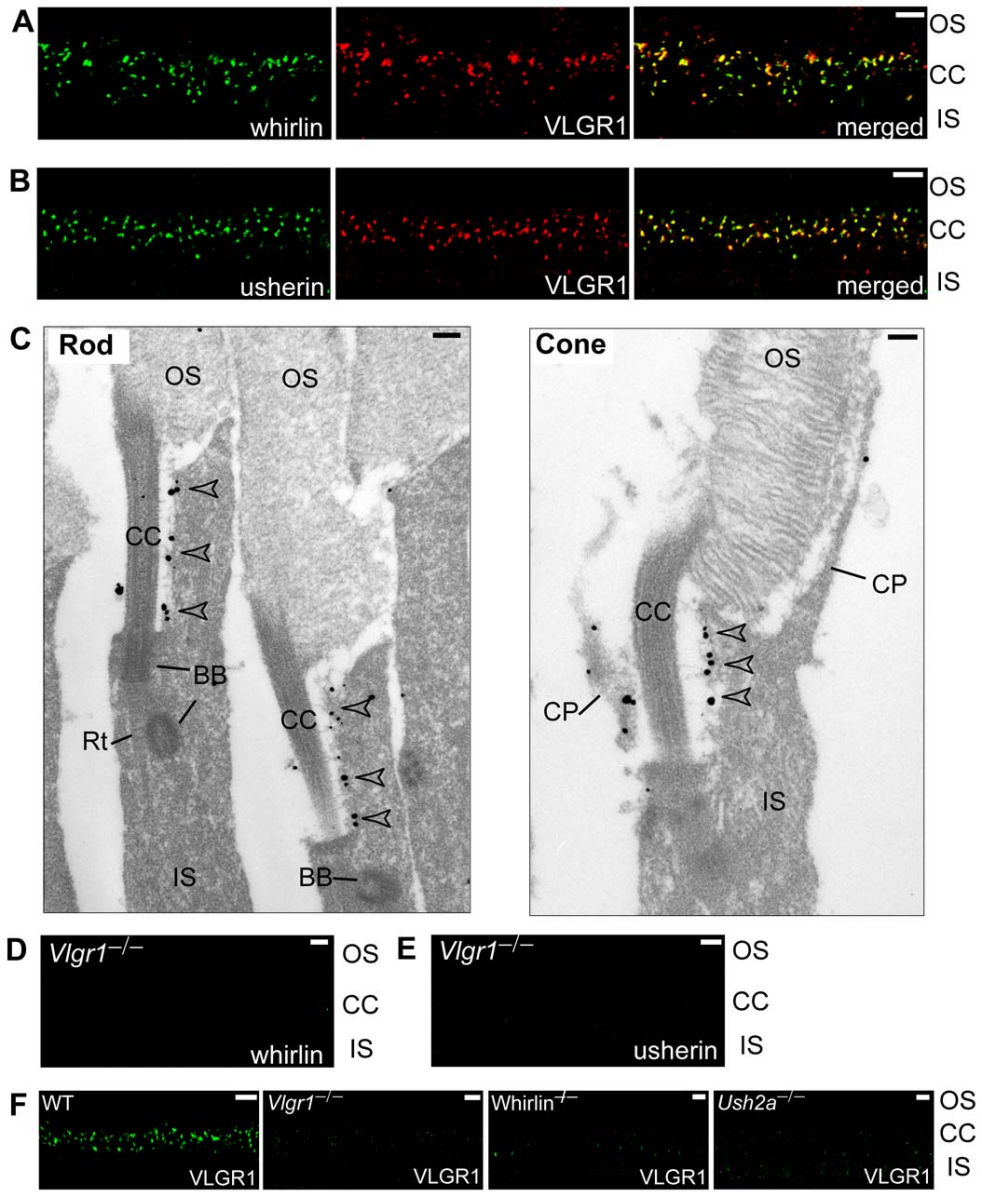
VLGR1 is colocalized with the complex of whirlin and usherin at the PMC in photoreceptors. VLGR1 (red) was colocalized with whirlin (green, A) and usherin (green, B) at the PMC in mouse photoreceptors. Immunoelectron microscopy (C) demonstrated that the gold labels of VLGR1 were present at the plasma membrane of the apical inner segment facing the connecting cilium, the PMC, in both mouse rod and cone photoreceptors (arrowheads). The signals of whirlin (D) and usherin (E) disappeared at the PMC in *Vlgr1* knockout photoreceptors. The signals of VLGR1 diminished in whirlin and *Ush2a* knockout mice (F). The staining of VLGR1 in wild-type (WT) and *Vlgr1* knockout mice serves as a positive and negative control, respectively. OS, outer segments; CC, connecting cilia; BB, basal bodies; Rt, the rootlet; IS, inner segments; CP, calycal processes. Scale bars, 5 µm (A,B,D–F) and 200 nm (C).

VLGR1 is the third known protein to be implicated in the USH2 etiology, and was previously reported to interact with whirlin *in vitro* [14]. Therefore, we studied whether VLGR1 was in the complex of whirlin and usherin in photoreceptors. Double staining of VLGR1 with either whirlin or usherin in the retina found VLGR1 to colocalize with both whirlin and usherin at the PMC in photoreceptors (Figure 6A and 6B). VLGR1 localization at the PMC in mouse rod and cone photoreceptors was further verified by immunoelectron microscopy (Figure 6C). Moreover, immunostaining demonstrated a decrease in VLGR1 signals at the PMC in whirlin and *Ush2a* knockout retina (Figure 6F), and an absence of whirlin (Figure 6D) and usherin (Figure 6E) proteins at the PMC in the *Vlgr1* knockout retina. These results indicate that whirlin, VLGR1 and usherin form a multi-protein complex *in vivo* at the PMC in photoreceptors and that functional deficits in any of these three known USH2 proteins destabilize this complex and disrupt its function.

### Whirlin, usherin, and VLGR1 are in the same multi-protein complex at the stereocilia of hair cells

**Figure 7:**
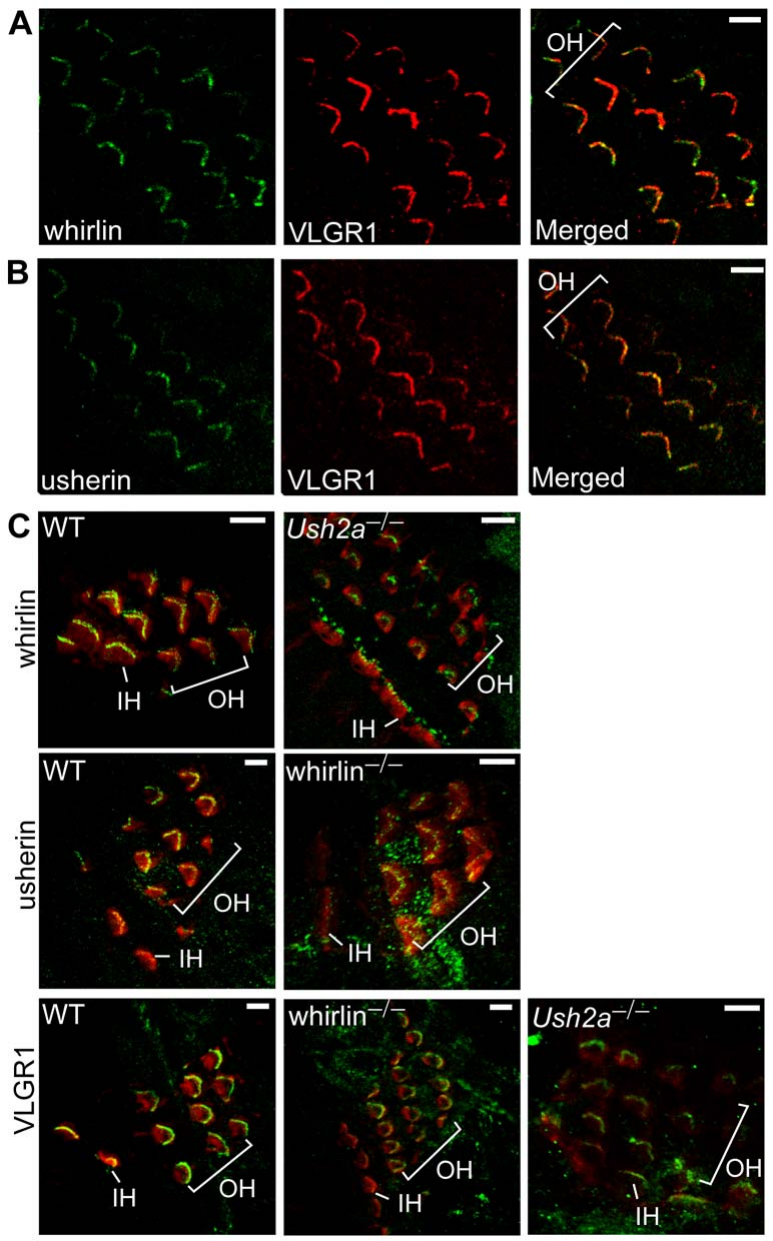
Whirlin, usherin and VLGR1 are colocalized in stereocilia of inner ear hair cells at postnatal day 4. Whirlin (green, A) and usherin (green, B) were colocalized with VLGR1 (red) in stereocilia in the mouse cochlea. Signals of whirlin (green, top row in C) and usherin (green, middle row in C) diminished in hair cell stereocilia (phalloidin, red) in the *Ush2a* and whirlin knockout cochleas, respectively. Signals of VLGR1 (green, bottom row in C) diminished at the hair cell stereocilia in both whirlin and *Ush2a* knockout cochleas. OH, outer hair cells; IH, inner hair cells. Scale bars, 5 µm.

Along the cochlear spiral, there are one row of inner hair cells and three rows of outer hair cells. The inner hair cells are responsible for mechanoelectric transduction, whereas the electromotile outer hair cells also perform an electromechanical transduction, thereby amplifying the sound-evoked vibrations of the entire sensory epithelium. Both types of hair cells have stereocilia on their apical surfaces, which are modified microvilli filled with bundles of actin filaments. The tips of the stereocilia are for the sites of the mechanoelectric transduction channels. Because of the involvement of USH2 proteins in hearing impairment in humans, we studied their localization in the cochlea. Double staining of the cochleas from mice aged at postnatal day (P) 3–6 showed VLGR1 colocalized with whirlin and usherin in the stereocilia bundles of both inner (data not shown) and outer hair cells (Figure 7A and 7B). The three USH2 proteins are localized to the ankle-link complex of the hair cell stereocilia [21]. This ankle-link complex appears as fine extracellular fibers at the base of the stereocilia bundle during development (P2–P12) [29]; however, its exact function is not clear. To study whether the three USH2 proteins are interdependent at the ankle-link complex as at the PMC, we examined their distribution in hair cells in whirlin and *Ush2a* knockout mice at P3–P6. The signals of whirlin and VLGR1 were decreased in *Ush2a* knockout mice and the signals for usherin and VLGR1 were decreased in whirlin knockout mice (Figure 7C). These data are consistent with the reported findings of mislocalization of USH2 proteins in whirler mice and one line of the *Vlgr1* mutant mice [21], and support the notion that whirlin, usherin and VLGR1 also form a multi-protein complex at the ankle-link complex of the stereocilia in hair cells, and the normal subcellular localizations of these three proteins are, to some extent, dependent on one another in the cochlea.

### Disruption of the N-terminal PDZ domains of whirlin leads to both vision and hearing defects in mice

**Figure 8:**
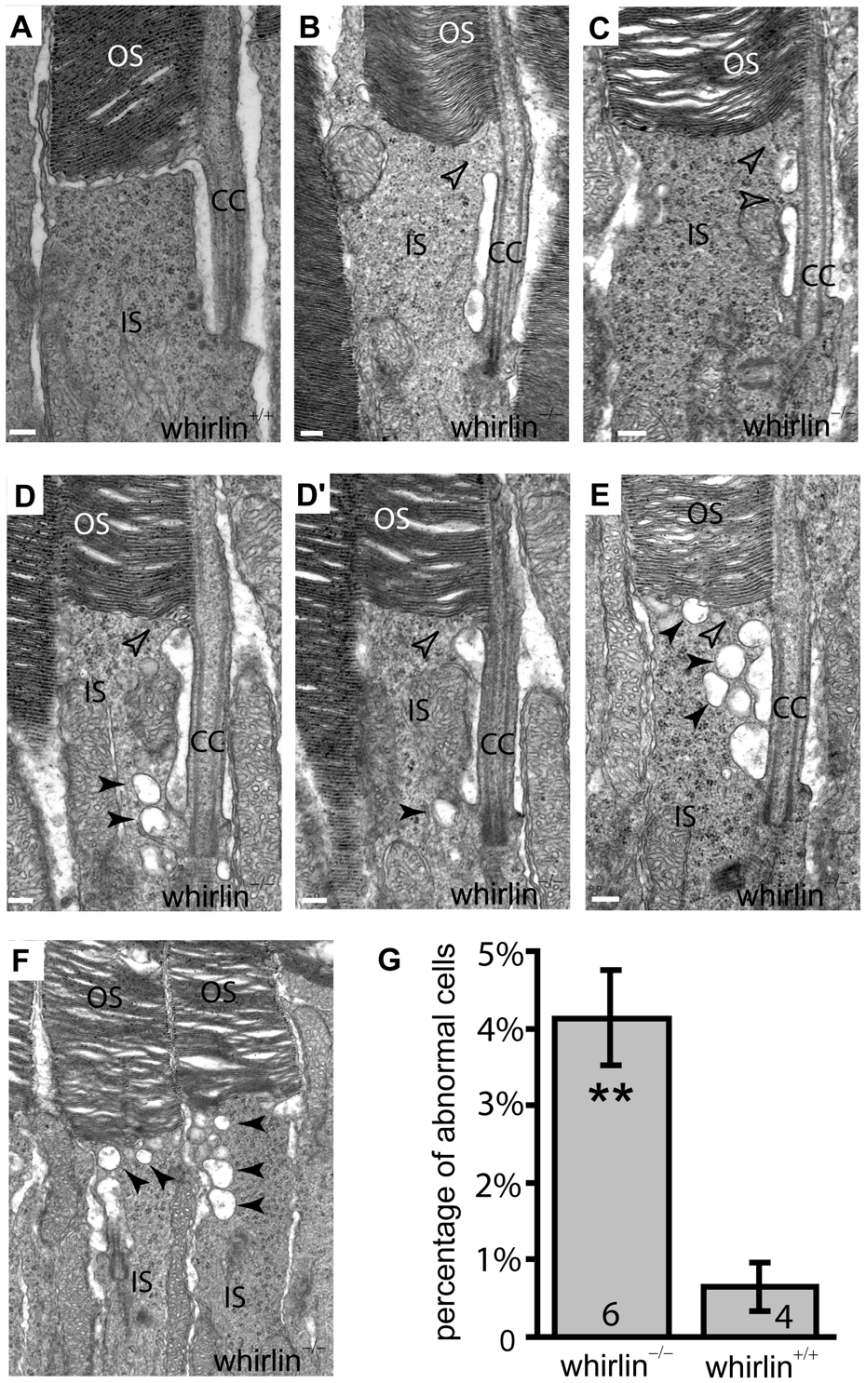
Whirlin knockout mice have morphological defects around the PMC in photoreceptors revealed by electron microscopy. (A) A representative image showing the normal ultrastructure around the PMC in the wild-type photoreceptor (whirlin^+/+^). (B–F) In whirlin knockout mice (whirlin^−/−^), abnormal distance (E) and membrane fusion (empty arrows, B–E) between the apical inner segment and the connecting cilium were found. In addition, a large amount of vacuoles (filled arrows, D–F) were accumulated around the PMC. (D,D′) show the same cell at different sectioning levels. OS, outer segments; CC, connecting cilia; IS, inner segments. Scale bars, 200 nm. (G) All the above abnormalities exist in a small fraction of photoreceptors randomly distributed in the retina of whirlin knockout mice. The number of mice analyzed in each group is indicated in the bottom of each bar. Mean ± SEM; **, p<0.01.

**Figure 9:**
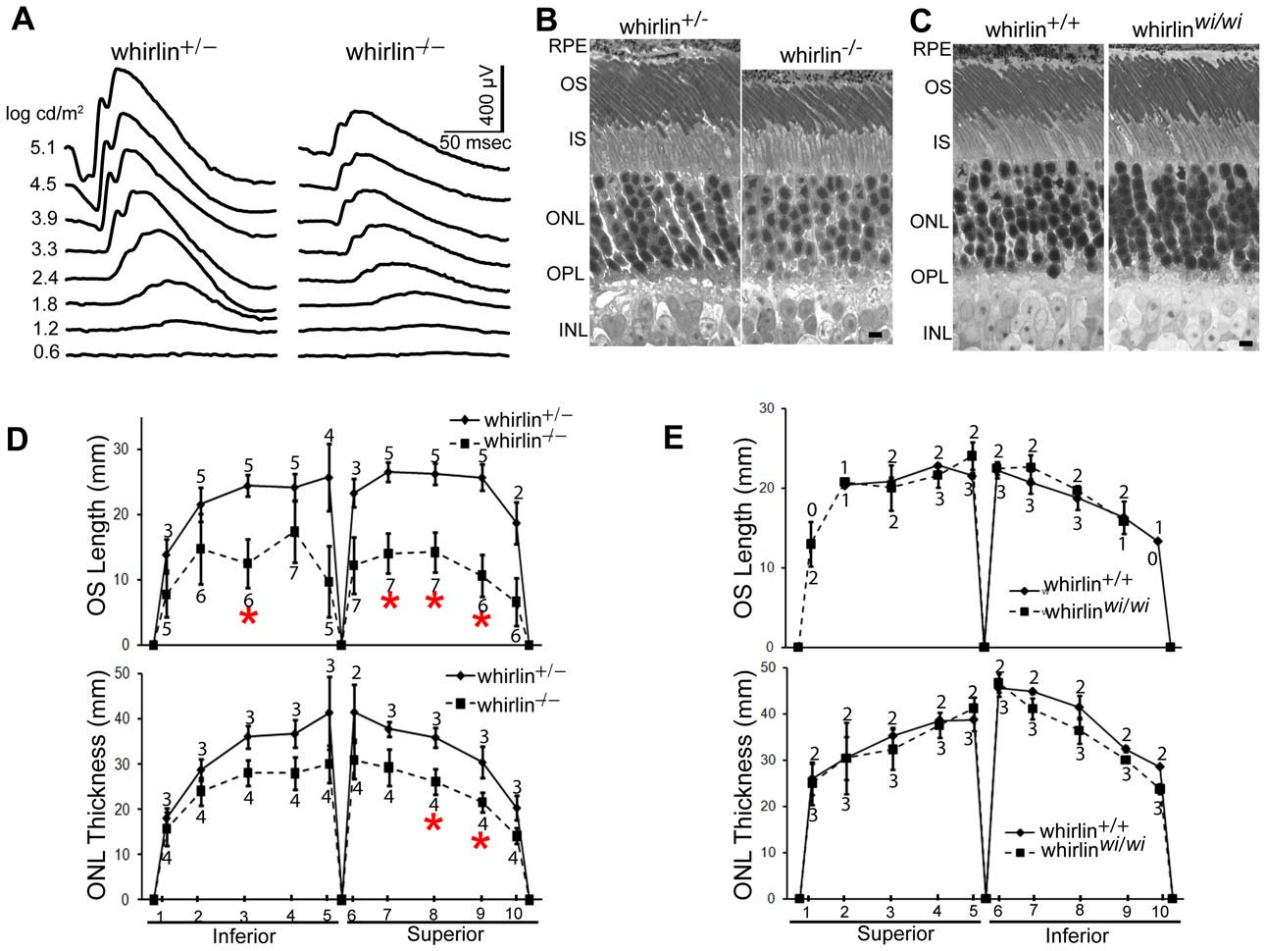
Retinal degeneration becomes apparent in whirlin knockout mice at 28–33 months of age. (A) Representative dark-adapted ERG tracings show reduced a- and b-waves in response to a series of white light stimuli in whirlin knockout mice at this age. (B) Representative 1-µm retinal sections stained with toluidine blue show shortened OS and reduced ONL in whirlin knockout mice. Scale bars, 5 µm. (C) Representative 1-µm retinal sections stained with toluidine blue show comparable thickness of OS and ONL between whirler mice and age-matched WT (whirlin^+/+^) mice. Scale bars, 5 µm. (D) Measurement of OS length and ONL thickness at different locations along the vertical meridian in whirlin knockout retinas. The number of mice measured at each location in each group is indicated above or below the lines. The error bar represents the standard error of the mean. *, p<0.05. Whirlin heterozygous littermates were used as a control in these studies. (E) Measurement of OS length and ONL thickness at different locations along the vertical meridian in whirler retinas. The numbers of mice measured at each point in whirler and age-matched wild-type cohorts are indicated below and above the lines, respectively. The error bar represents the standard error of the mean. RPE, retinal pigment epithelium; OS, outer segment; IS, inner segment; ONL, outer nuclear layer; OPL, outer plexiform layer; INL, inner nuclear layer.

Retinal function tested by electroretinogram (ERG), a recording of the retinal electrical response to flashes of light, and histology examined by light microscopy did not reveal overt retinal degeneration in whirlin knockout mice up to 24 months of age (data not shown). However, morphological defects were evident at the ultrastructural level as early as 5 months of age. Examination by electron microscopy found membrane fusion between the apical inner segment and the connecting cilium and accumulation of vacuoles next to the PMC in the apical inner segment (Figure 8). The synaptic terminus of photoreceptors appeared normal (data not shown). Further analysis of whirlin knockout mice aged from 28 to 33 months found that the amplitudes of both a- and b-waves of dark-adapted ERG recordings significantly decreased compared to their heterozygous littermate controls. The light-adapted ERG amplitudes also decreased although the difference did not reach statistical significance (Figure 9A and Table 1). Histological examination of the eyes from this cohort of animals found that the photoreceptor nuclear layer was significantly thinner and the outer segments shortened in the whirlin knockout mice (Figure 9B and 9D), which are signs for retinal degeneration. Thus both functional and morphological assays indicate that the whirlin knockout mice develop late-onset retinal degeneration. In contrast, histological examination of the whirler mouse retina from 28 to 33 months of age did not find any abnormalities compared with age-matched wild-type controls (Figure 9C and 9E).

**Table 1:** ERG analysis in whirlin knockout mice at 28–33 months of age.

	Control	whirlin^−/−^
**ERGs**		
**Dark-adapted**		
**a-wave amplitude (µV)**	169.6±10.6, 4	82.4±14.2, 7 (<0.004)
**b-wave amplitude (µV)**	570.3±47.9, 4	318.7±45.4, 7 (<0.004)
**Light-adapted**		
**b-wave amplitude (µV)**	71.7±11.0, 4	47.1±8.2, 7

Values are given as mean ± SEM, n (p). n denotes the number of animals analyzed. p values are given where significant.

**Figure 10:**
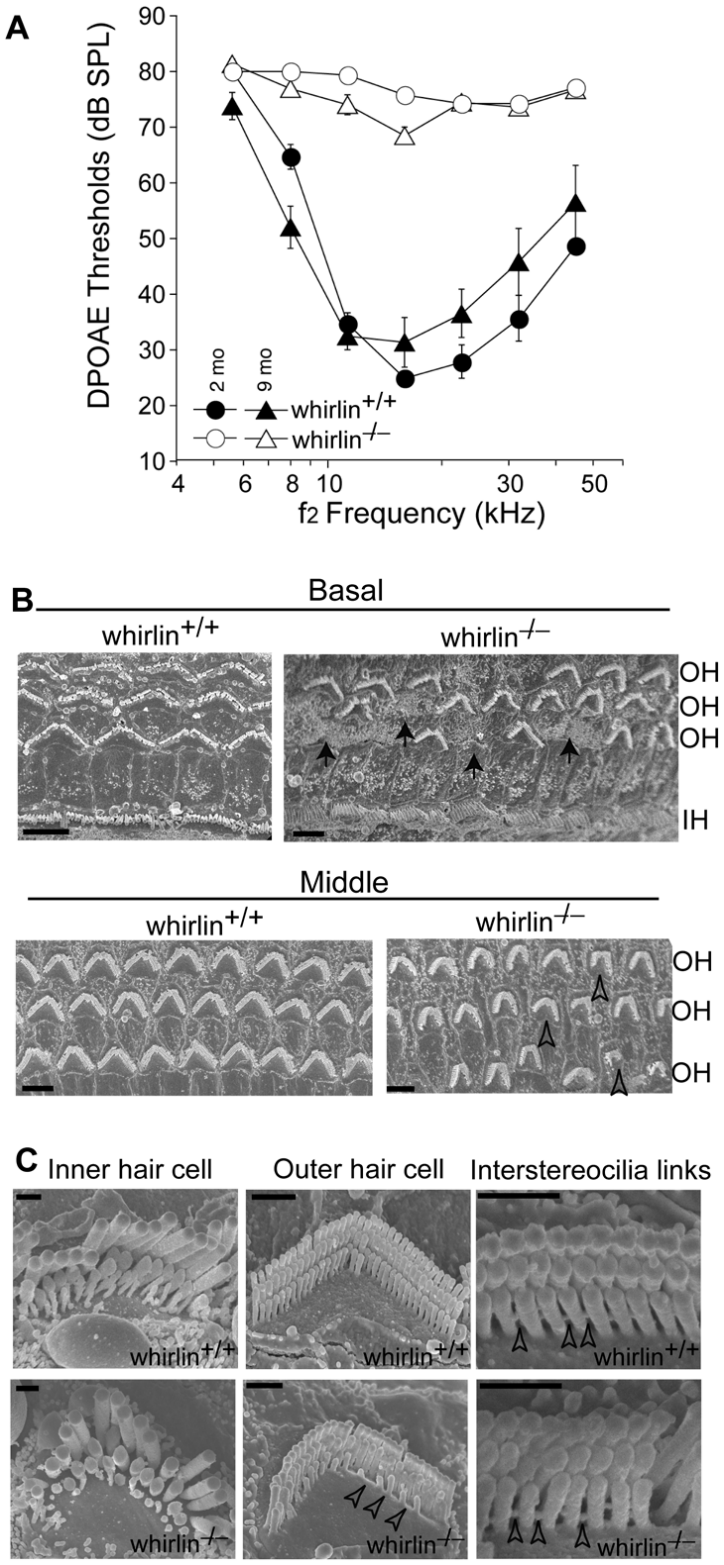
Whirlin knockout mice have non-progressive hearing defects. (A) DPOAE analysis demonstrates that whirlin knockout mice have profound hearing loss at all stimulus frequencies, as measured at either 2 or 9 months of age. (B) Scanning electron microscopy shows scattered loss of outer hair cells (arrows) in the basal turn and dysmorphology of the stereocilia bundles in all cochlear regions (e.g. open arrowheads) in whirlin knockout mice. (C) At high magnification, in whirlin knockout mice, the inner hair cell stereocilia appear normal (left column); the outer hair cells show patchy loss of stereocilia in the innermost (shortest) row of the hair bundle (arrowheads, middle column); the interstereocilia links of the outer hair cells appear normal (arrowheads, right column). OH, outer hair cells; IH, inner hair cells. Scale bars, 5 µm (B) and 1 µm (C).

We measured distortion product otoacoustic emissions (DPOAE) to assay cochlear function in two groups of whirlin knockout mice at 2 and 9 months of age, respectively (Figure 10A). At both ages, knockouts showed no cochlear responses (i.e. thresholds were above the measurement ceiling at which the system produces its own distortion components), thus demonstrating a profound congenital hearing loss across all cochlear frequencies. Light microscopic evaluation of whirlin knockout ears at 2 months of age (data not shown, n = 2) showed only sporadic loss of hair cells in the mid-basal turn. All other accessory structures of the inner ear, including spiral ligament and stria vascularis, appeared normal. There did not appear to be a substantial loss of cochlear neurons. Scanning electron microscopy was performed to examine the morphology of cochlear stereocilia. Throughout the cochlear spiral, hair bundles on outer hair cells were abnormally compressed in the spiral dimension, i.e. the angle between the two limbs of the “V” shaped formation was smaller in the knockout ears (Figure 10B). In general, the hair bundle formation exhibits a “U” shape, which is a morphological defect characteristic of USH2 mutant mice [21], [26], [30]. Closer examination (Figure 10C) showed that, although some outer hair cell stereocilia bundles were normal with obvious interstereocilia links, many showed a patchy loss of stereocilia from the innermost (shortest) row of stereocilia. The inner hair cell stereocilia were normal in appearance throughout the cochleas. Given the critical role of outer hair cells in cochlear amplification and the production of DPOAEs, these stereocilia abnormalities in whirlin knockouts could explain the cochlear dysfunction.

## Discussion

**Figure 11:**
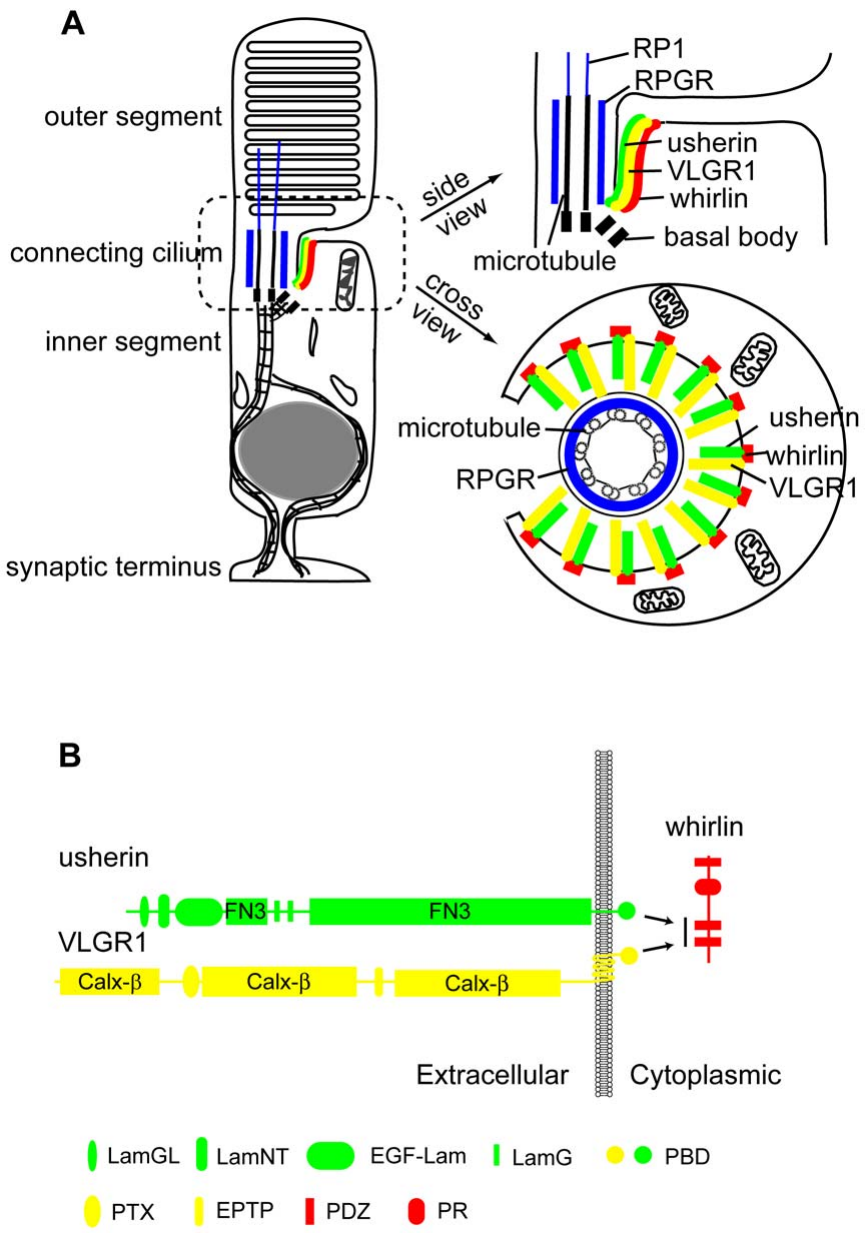
Schematic diagrams illustrate the USH2 multi-protein complex at the PMC in photoreceptors. (A) Localization of the complex of whirlin (red), usherin (green) and VLGR1 (yellow) at the PMC in mammalian photoreceptors. On the left is a whole-cell view. On the right are the enlarged longitudinal and cross-sectional views. (B) The interactions among whirlin, usherin and VLGR1. PR, proline-rich region; LamGL, LamG-like jellyroll fold domain; LamNT, laminin N-terminal domain; EGF-Lam, laminin-type epidermal growth factor-like domain; FN3, fibronectin type 3 domain; LamG, laminin G domain; PBD, PDZ-binding motif; Calx-β, domains in Na-Ca exchangers and integrin-beta4; PTX, pentraxin domain; EPTP, epitempin domain.

The whirlin knockout mice characterized in this study have a late-onset retinal degeneration and a congenital, non-progressive hearing impairment. This phenotype reiterates the clinical features of USH2D disease in humans [12]. Therefore, this whirlin knockout mouse line is an appropriate animal model for studying the pathogenesis of this disease. In this study, we have provided definitive evidence on the *in vivo* interaction of whirlin with usherin and VLGR1 in both the retina and the inner ear. Because these three proteins are all involved in USH2, this finding suggests that the USH2 proteins function coordinately as a multi-protein complex *in vivo*. Usherin and VLGR1 are proteins with an extremely large extracellular region containing multiple repeats of a number of known cell adhesion motifs. It is believed that usherin and VLGR1 may participate in the linkage to various extracellular matrix proteins and/or cell adhesion proteins. Thus, it is essential that they be anchored at specific plasma membrane microdomains of the cells to fasten these linkages. Their interaction with whirlin appears to provide this anchorage by binding them to a submembrane protein supramolecular complex. Moreover, the localization of the protein supramolecular complex at the plasma membrane of the PMC requires binding of whirlin to usherin and VLGR1. As a result, the three proteins are interdependent for their normal subcellular localization and stability in photoreceptors (Figure 11) and hair cells. In the absence of one USH2 protein, the other two USH2 partner proteins are dispersed and destabilized, and are presumed to no longer function normally. This observation has important implications for understanding the disease mechanisms of USH2. First, all three USH2 subtypes, despite their genetic heterogeneity, affect the same subcellular target in photoreceptors and hair cells. Second, loss of one USH2 gene function is predicted to be functionally close to loss of all three. Third, the photoreceptor degeneration in USH2 disease arises from dysfunction of the PMC, a subcellular structure that is conserved from amphibian to mammalian photoreceptors.

The PRC, the analogous structure of the PMC in frog photoreceptors, is a set of nine symmetrically arrayed ridges and grooves, seen by scanning electron microscopy, at the apical inner segment membrane surrounding the connecting cilium. Originally discovered over 20 years ago [31], [32], the molecular components of the PRC had remained unknown. In the present study, we show that whirlin is a component of the PRC, the first identified marker for this complex in frogs. Although a morphologically apparent PRC structure in mammalian photoreceptors has not been seen, the similar localization pattern of whirlin in frog and mouse photoreceptors strongly suggests that a functional equivalent structure of the PRC exists in mammalian photoreceptors. Hence, we propose that the mammalian equivalent of the PRC be designated the periciliary membrane complex (PMC). Our group was the first to propose the concept of a PRC equivalent structure in mammalian photoreceptors based on the subcellular distribution of whirlin [33]. Our findings in the present study of the subcellular localization and functional interaction among whirlin, usherin and VLGR1 in mouse photoreceptors further strengthen this argument. In frogs, numerous rhodopsin-containing vesicles are present in the surrounding cytoplasm of the PRC, suggesting that the PRC may be a docking site of vesicles transporting newly synthesized rhodopsin from the Golgi [32]. Consistent with this theory, we found accumulation of vacuoles around the PMC in a small proportion of photoreceptors in whirlin knockout mice. However, in both whirlin knockout and *Ush2a* knockout mice [26], polarized distribution of rhodopsin to the outer segments was not measurably disrupted as shown by immunofluorescence. This observation suggests that either loss of these proteins is not sufficient to abolish completely the organization and function of the PMC, or alternative routes exist in mammalian photoreceptors for targeting rhodopsin to the outer segments. It is also possible that the USH2 protein complex is not involved directly in protein trafficking but plays only a structural role. Interestingly, we have found that the spacing between the PMC and the connecting cilium became irregular in whirlin knockout mice and there was frequent occurrence of membrane fusion between the PMC and the connecting cilium. These findings indicate that the USH2 proteins are important in maintaining the integrity of the spatial relationship between the PMC and the juxtaposing connecting cilium.

A series of *in vitro* analyses have found that whirlin is able to interact with calmodulin-dependent serine kinase [34], NGL-1 [24], SANS [23], myosin VIIa [24] as well as usherin [17] and VLGR1 [14]. Among these proteins, SANS and myosin VIIa are involved in human Usher syndrome type I [20], [35]. They have been reported to localize at or in the vicinity of the connecting cilium in photoreceptors [23], [36]. In inner ear hair cells, immunostaining, biochemical and cellular analyses suggest that the interaction between whirlin and myosin XVa through the PDZ domains of whirlin is required for delivery of whirlin to the tip of stereocilia [13], [24], [37]. Additionally, whirlin has been shown to interact with p55 in hair cells [38]. Therefore, some of these proteins might be candidate components of the PMC, although further studies are necessary to verify their presence in the PMC *in vivo*, Such studies could lead to a more comprehensive understanding of this specialized membrane domain.

In the inner ear hair cells, the interaction among whirlin, usherin and VLGR1 plays a similar role in localizing the USH2 protein complex at their normal subcellular location, i.e., the stereocilia. Here, the interactions among the three proteins may be subtly different from those in photoreceptors. The three proteins may not be completely dependent on one another for their normal localization in hair cells, as indicated by the incomplete loss of the complex from stereocilia in whirlin and *Ush2a* single knockouts. Usherin and VLGR1 have been demonstrated in this study and a recent study [14] to bind to the N-terminal two PDZ domains in whirlin. In the inner ear, the high abundance of whirlin short isoform, which lacks these two PDZ domains, may make the interaction of whirlin long isoform with usherin and VLGR1 partially redundant. Additionally, there may be different proteins participating in the formation and localization of the multi-protein complex containing whirlin, usherin and VLGR1 between photoreceptors and hair cells. For example, a unique exon in the cytoplasmic region of usherin in hair cells, which is missing in photoreceptors [17], may provide a platform for binding yet unidentified proteins in hair cells.

In contrast to previous studies on the localization of USH2 proteins in photoreceptors [14], [22], [23], we localized whirlin and VLGR1 only to the PMC in photoreceptors as what we have found for usherin in one of our recent publications [26]. To further confirm this finding, we rigorously exploited two approaches, double immunostaining of whirlin with different subcellular structure markers in two different species and immunoelectron microscopy. We determined the specificity of our antibodies of USH2 proteins in western blotting and immunostaining using *USH2* mutant mice as valid negative controls. Additionally, ultrastructural examination of whirlin knockout mice found various defects only around the PMC but not in other regions, such as the synaptic terminus, in photoreceptors. Therefore, our study presents strong evidence that the USH2 proteins are only located at the PMC in photoreceptors.

Comparison of whirlin knockout mice generated in this study with the whirler mice demonstrates that whirlin long isoform plays an essential role in photoreceptors. In our whirlin knockout mice, whirlin long isoform including the first and second PDZ domains, which bind to usherin, have been disrupted. By immunofluorescence, usherin is lost from the PMC (Figure 5B). Since usherin is required for maintaining the long term viability of photoreceptors [26], the absence of usherin from the PMC could be responsible, at least in part, for the late-onset retinal degeneration in whirlin knockout mice. In whirler mice, a large deletion in the whirlin gene (Figure 1A) removes all predicted translational start codons of the short isoform and a portion of the proline-rich region. This mutation, therefore, is believed to completely ablates the short isoform and truncates the long isoform leaving only the N-terminal PDZ domains intact. Furthermore, an N-terminal whirlin transcript that we have found in abundance by cDNA library screening is predicted to produce a protein that retains the first PDZ domain. These N-terminal whirlin protein variants appeared to partially compensate for the loss of the intact whirlin long isoform. Indeed, in whirler mice a reduced amount of usherin is still found at the PMC in photoreceptors (Figure 5B). This residual whirlin/usherin function appears to be sufficient in maintaining photoreceptor viability, and hence no photoreceptor degeneration was found in whirler mice. Our finding that whirlin long isoform protein alone is expressed in the retina further supports the notion that the long but not the short variant of whirlin is required in photoreceptors.

The differences in hearing and vestibular dysfunction and in hair cell stereocilia defects between whirlin knockout and whirler mice suggest that whirlin long and short isoforms may function differently in hair cells. In whirlin knockout mice, only the outer hair cell stereocilia exhibit an abnormal ‘U’ shape formation, while the inner hair cell stereocilia appear normal. These whirlin knockout mice are partially deaf and have no circling behavior (no vestibular defect). But in whirler mice, besides the abnormal ‘U’ shape stereocilia formation in the outer hair cells, the inner hair cells have significantly shortened stereocilia [10], [39], [40]. These mice are completely deaf and exhibit a vestibular balance problem.

The position-dependent outcome of whirlin gene mutations observed in mice is also apparent in humans. In a German USH2 family, compound heterozygosity of a nonsense mutation p.Q103X and a mutation in the splice donor site, c.837+1G>A, which are in the 5′-terminal region of the whirlin gene, was found to cause USH2 [12]. In addition, a nonsense mutation, p.R778X, and a single nucleotide deletion, c.2324delG, leading to a deletion of the C-terminus of the whirlin protein were found responsible for deafness DFNB31 [10], [11]. Therefore, in both humans and mice, mutations at the N-terminus of the whirlin protein cause both vision and hearing impairments (our study and [12]), while mutations at the C-terminus of the whirlin protein cause more severe hearing defects only [10], [11]. These data support our conclusion that the long isoform plays an essential role in photoreceptors, while the short isoform functions primarily in hair cells. In summary, this study provides strong evidence that USH2 proteins form a multi-protein complex in which the whirlin long isoform plays a key role. This complex is localized at the PMC in photoreceptors and the stereocilia in hair cells. Disruption of this USH2 protein complex could be the common pathogenic mechanism underlying all three subtypes of human USH2 disease.

## Materials and Methods

### Generation of whirlin knockout mice

Two genomic DNA fragments (2.8 and 6 kb) flanking the 3′ portion of the first exon of whirlin were amplified from 129/Sv mouse genomic DNA by PCR, and inserted separately as the short and long arm into a modified pGT-N29 vector, which contained a diphtheria toxin expression cassette as a negative selection marker (Figure 1B). The targeting vector was linearized and electroporated into R1 embryonic stem (ES) cells. An ES clone was found to have the partial replacement of the first exon of whirlin by the Neo^r^ gene, and was microinjected into C57BL/6 blastocysts. The resulting chimeras were crossed with C57BL/6 mice. Heterozygous and homozygous knockout mice were identified with respect to the targeted allele by PCR (Figure 1C). The MEEI institutional guidelines were followed on all animal procedures.

### Isolation of genomic DNA and total RNA, PCR, and RT–PCR reactions

A tiny piece of the mouse tail (about 2 mm long) was lysed by proteinase K at 50°C overnight in tissue lysis buffer (100 mM Tris-HCl pH 8.0, 200 mM NaCl, 5 mM EDTA, and 0.2% SDS). The genomic DNA was precipitated from the resulting lysate by adding the same volume of isopropanol and centrifugation. The pellet was finally dissolved in TE buffer. The total RNA was isolated using TRIzol Reagent (Invitrogen) according to the manufacturer's instruction. RT (ThermoScript™ RT-PCR system, Invitrogen) and PCR (Expand long template PCR system, Roche Diagnostics) reactions were performed following the manufacturer's instructions.

### Antibodies

Mouse whirlin cDNA fragments (PDZ320, 1–320 aa; PDZIE, 315–580 aa, accession number, NP_082916) and frog whirlin cDNA fragment (analogous to mouse whirlin 816–907 aa) were inserted into the expression vector pET28 (Novagen). Recombinant proteins were expressed as His-tagged fusion proteins in *Escherichia coli* host BL21-CodonPlus (DE3)-RIPL. The recombinant proteins were purified through a Ni^2+^-charged nitriloacetic acid agarose column and were used to immunize rabbits and chickens. Whirlin-specific antibodies were affinity-purified from antisera or egg yolk extracts. Usherin antibodies used in this study were raised against the N-terminal and C-terminal domains of the protein [26]. RP1, RPGR and CIP98 antibodies were as described previously [25], [34], [41], [42]. VLGR1 antibody was kindly provided by Dr. Perrin C. White (University of Texas Southwestern Medical Center, Dallas, Texas). Mass1 (C20) antibody was purchased from Santa Cruz Biotechnology, Inc. Monoclonal anti-γ-tubulin and anti-acetylated α-tubulin antibodies were obtained from Sigma-Aldrich. Alexa fluorochrome-conjugated phalloidin and secondary antibodies, and Hoechst dye 33342 were obtained from Molecular Probes, Inc.

### Yeast two-hybrid analysis

Mouse whirlin and its fragments (full-length, 3–907 aa; N-terminus, 3–472 aa; C-terminus, 438–907 aa, accession number, NP_082916) and mouse usherin fragment (5053–5193 aa, accession number, NP_067383) were amplified from the retina and individually cloned into both pGBKT7 and pGADT7 vectors. Yeast two-hybrid analysis was performed as described previously [43]. Briefly, a protein/peptide in pGBKT7 vector was co-transformed with its putative interacting protein/peptide in pGADT7 vector. Empty pGBKT7 and pGADT7 vectors were used as negative controls. Co-transformants were grown on both SD-4 (-Leu, -Trp, -Ade, and -His) and SD-2 (-Leu and -Trp) plates. The growth on SD-4 plates indicated an existence of interaction between the two co-transformed proteins/peptides. In our experiments, all co-transformants were able to grow on SD-2 plates indicating a successful co-transformation.

### GST pull-down assay, immunoprecipitation, and western blotting

GST pull-down assay: cDNA fragments of intact (mouse: 5053–5193 aa, NP_067383; frog: analogous to mouse usherin 5053–5193 aa) and mutant (without PDZ-binding domain, mouse: 5053–5186 aa, NP_067383; frog: analogous to mouse usherin 5053–5189 aa) C-terminal usherin were amplified from frog and mouse retinas and cloned into the pGEX4T-1 vector. The GST-fused intact and mutant usherin were expressed in BL21-CodonPlus (DE3)-RIPL cells and then incubated with mouse retinal lysate and glutathione sepharose beads for 2 hours at 4°C. Subsequently, the sepharose beads were washed with lysis buffer (50 mM Tris-HCl pH8.0, 150 mM NaCl, 0.5% TritonX-100, 5 mM EDTA, 0.5 mM PMSF, 1× protease inhibitor, and 1 mM DTT) for three times and boiled in Laemmli sample buffer for 10 minutes. All the procedures were performed at 4°C. Retinal lysates incubated with glutathione sepharose beads and GST or only with GST were used as negative controls.

Immunoprecipitation: Dissected retinal or inner ear tissues were homogenized and incubated for about 60 minutes in lysis buffer. After centrifugation at 18,000 g for 10 minutes, the supernatants were precleared by incubation with protein G sepharose (Amersham Biosciences) for 1 hour. Subsequently, they were incubated with the primary antibodies for 3 hours and then centrifuged at 18,000 g for 10 minutes. The resulting supernatants were incubated with protein G sepharose for an additional 1 hour. After a brief centrifugation at 2000 g, the pellets were washed with lysis buffer for four times and then boiled in Laemmli sample buffer. All the procedures were performed at 4°C. A non-immune rabbit IgG served as a negative control. Western blotting was carried out as described previously [43].

### Immunofluorescence, immunoelectron microscopy, transmission electron microscopy, and scanning electron microscopy

Immunofluorescence: Eyes were enucleated, frozen immediately and sectioned at 10-µm thick. Sectioned tissues were fixed in 4% formaldehyde/PBS for 10 minutes (for usherin staining, 2% formaldehyde/PBS for 5 minutes), and permeabilized by 0.2% Triton X-100/PBS for 5 minutes at room temperature. Pup heads on postnatal day 3–6 were fixed in 4% formaldehyde/PBS for about 36 hours, switched to 30% sucrose/PBS for several days, and sectioned at 30-µm thick. The subsequent steps of blocking and incubation with primary and secondary antibodies were as described previously [43]. Alexa 488- and 594-conjugated secondary antibodies were routinely used for tissue double-labeling. Stained sections were viewed and photographed on a fluorescent microscope (Olympus, model 1X70) equipped with a digital camera (Carl Zeiss MicroImaging, Inc.) or on a confocal laser scanning microscope (Leica, model TCS SP2).

Immunoelectron microscopy: Eyes were enucleated. Their anterior segments and lens were removed. Dissected retina was fixed with 4% formaldehyde/PBS (whirlin) or 2% formaldehyde/0.1% glutaraldehyde/PBS (VLGR1) for 30 minutes, washed with TTBS buffer (Tween/Tris-buffered saline), blocked in 5% goat serum/TTBS for 1 hour, and incubated with the primary antibodies at 4°C overnight. After rinses, the retina was incubated with Nanogold goat anti-rabbit antibody (Aurion, Wageningen, The Netherlands), post-fixed sequentially with 1% formaldehyde/2.5% glutaraldehyde/0.1 M cacodylate buffer and 2% osmium tetroxide. Later, it was silver-enhanced, dehydrated, embedded in Epon, and sectioned at 70 nm thickness. In an alternative protocol, the retina was fixed in 2% formaldehyde/0.1% glutaraldehyde/PBS for 30 minutes, frozen and cut to 10 µm sections prior to staining with primary and secondary antibodies. Staining was done while floating in a dish. After final wash, the sections were post-fixed and processed for immunoEM as above. ImmunoEM for whirlin were studied with both methods which yielded the same results. ImmunoEM for VLGR1 used the alternative protocol.

Transmission electron microscopy and scanning electron microscopy was performed as described previously [26], [43].

### Morphological analyses of the retina

Measurements of photoreceptor outer segment length and outer nuclear layer thickness were made along the vertical meridian (superior to inferior) at five locations to each side of the optic nerve head separated by approximately 600 µm each. Measurements began at approximately 200 µm from the optic nerve head and ended at approximately 200 µm from the retinal periphery. For the analysis in the whirlin knockout mice, seven whirlin knockouts and five whirlin heterozygous littermates from 28–33 months of age were included. For the analysis in the whirler mice, three whirler mice aged from 28–33 months and two age-matched wild-type mice were included.

Photoreceptors with abnormal morphology around the PMC were counted at the retinal mid-periphery. Abnormal morphology was defined as membrane fusion between the apical inner segment and the distal connecting cilium or vacuole accumulation in the apical inner segment around the PMC. The presence of at least 3 large vacuoles (diameter is larger than 200 nm) or 4 small vacuoles (diameter is about 100 nm) was considered as vacuole accumulation. Four wild-type and six whirlin knockout mice at the age from 5 to 24 months were included in this experiment.

The Student's *t*-test was conducted to compare the measured values of whirlin knockout and control mice. A *P* value of less than 0.05 was considered to indicate a significant difference between the two groups.

### ERG and DPOAE measurements

ERG and DPOAE recordings were performed as described previously [26], [44].
